# β-glucuronidase use as a single internal control gene may confound analysis in *FMR1* mRNA toxicity studies

**DOI:** 10.1371/journal.pone.0192151

**Published:** 2018-02-23

**Authors:** Claudine M. Kraan, Kim M. Cornish, Quang M. Bui, Xin Li, Howard R. Slater, David E. Godler

**Affiliations:** 1 Cyto-molecular Diagnostic Research Laboratory, Victorian Clinical Genetics Services and Murdoch Children’s Research Institute, Royal Children’s Hospital, Melbourne, Victoria, Australia; 2 School of Psychological Sciences and Monash Institute of Cognitive and Clinical Neurosciences, Monash University, Clayton, Victoria, Australia; 3 Department of Paediatrics, The University of Melbourne, Melbourne, Victoria, Australia; 4 Centre for Epidemiology and Biostatistics, University of Melbourne Carlton, Victoria, Australia; University of Minnesota Duluth, UNITED STATES

## Abstract

Relationships between Fragile X Mental Retardation 1 (*FMR1*) mRNA levels in blood and intragenic *FMR1* CGG triplet expansions support the pathogenic role of RNA gain of function toxicity in premutation (PM: 55–199 CGGs) related disorders. Real-time PCR (RT-PCR) studies reporting these findings normalised *FMR1* mRNA level to a single internal control gene called *β*-glucuronidase (*GUS)*. This study evaluated *FMR1* mRNA-CGG correlations in 33 PM and 33 age- and IQ-matched control females using three normalisation strategies in peripheral blood mononuclear cells (PBMCs): (i) *GUS* as a single internal control; (ii) the mean of *GUS*, Eukaryotic Translation Initiation Factor 4A2 *(EIF4A2*) and succinate dehydrogenase complex flavoprotein subunit A (*SDHA*); and (iii) the mean of *EIF4A2* and *SDHA* (with no contribution from *GUS*). *GUS* mRNA levels normalised to the mean of *EIF4A2* and *SDHA* mRNA levels and *EIF4A2*/*SDHA* ratio were also evaluated. *FMR1*mRNA level normalised to the mean of *EIF4A2* and *SDHA* mRNA levels, with no contribution from *GUS*, showed the most significant correlation with CGG size and the greatest difference between PM and control groups (*p* = 10^−11^). Only 15% of *FMR1* mRNA PM results exceeded the maximum control value when normalised to *GUS*, compared with over 42% when normalised to the mean of *EIF4A2* and *SDHA* mRNA levels. Neither *GUS* mRNA level normalised to the mean RNA levels of *EIF4A2* and *SDHA*, nor to the *EIF4A2/SDHA* ratio were correlated with CGG size. However, greater variability in *GUS* mRNA levels were observed for both PM and control females across the full range of CGG repeat as compared to the *EIF4A2/SDHA* ratio. In conclusion, normalisation with multiple control genes, excluding *GUS*, can improve assessment of the biological significance of *FMR1* mRNA-CGG size relationships.

## Introduction

The prevalence of *FMR1* Premutation (PM: CGG 55–199) alleles in the general population has been reported to be as high as 1 in 150 females and 1 in 450 males [1]. Maternally transmitted PM alleles have the propensity to expand in future generations to full mutation (CGG ≥200) alleles that cause fragile X syndrome (FXS) [2]. FXS is a common cause of intellectual disability and co-morbid autism (reviewed in [3]). PM alleles have also been associated with adult onset Fragile X-associated disorders: Fragile X-associated tremor/ataxia syndrome (FXTAS: 40% males and 8–16% females over 50 years old) and Fragile X-associated primary ovarian insufficiency (FXPOI: ~20% females) [4, 5]. Pathogenic mechanisms suggested to cause PM related disorders include reduced *FMR1* protein expression (FMRP), elevated levels of non-coding RNA (*FMR4*, *FMR5*, *FMR6*) [6, 7], mitochondrial dysfunction [8] and CGG repeat-associated non-AUG translation [9]. The mechanism most extensively studied in the context of Fragile X-associated disorders is *FMR1* mRNA gain of function toxicity [10].

*FMR1* mRNA levels in the blood have been reported to be elevated 2–8 fold in PMs as compared to individuals with normal *FMR1* alleles (<45 CGG repeats). This finding has been replicated across many different cell types in humans and in various CGG knock-in animal models [10, 11]. It is thought that this PM-specific *FMR1* mRNA excess is indirectly associated with increase in CGG size in the PM range and the formation of intranuclear inclusion bodies and late-onset neurodegeneration [12]. Indeed, PM-size ribo-rCGG repeat containing *FMR1* mRNA can induce formation of intranuclear inclusions in Purkinje neurons of the cerebellum in transgenic mice, also known as nuclear foci [13]. Moreover, inclusion bodies that stain positive for *FMR1* mRNA have been found in both the central nervous system of men with FXTAS [14–16] and in mice with ‘knock in’ PM alleles [17, 18].

*FMR1* mRNA levels in peripheral tissues have been significantly correlated with brain changes associated with FXTAS and with subtle motor signs in adult PM carriers at risk for FXTAS [19–21]. However, other studies failed to identify any relationship between the *FMR1* mRNA level and similar clinical outcome measures, even in cases where relationships between the phenotype and CGG size were present [22, 23].

The first study to propose the RNA toxicity mechanism used real-time PCR, normalising *FMR1* mRNA levels with *GUS* as a single internal control [10]. This study reported that normal peripheral blood leukocytes maintain comparable levels of *FMR1* and *GUS* mRNA, although no data was presented examining variability of *GUS* mRNA level as compared to other internal control genes in the control and PM groups.

While *GUS* is commonly used as an internal control gene for RT-PCR normalisation across different settings, especially in plant biology studies, its transcription stability in mammalian systems has been variable between studies and cell types [24–28]. Analysis of *GUS* mRNA stability as compared to other genes in lymphoid malignancies or B and T cell enriched and stimulated leukocyte fractions found that *GUS* had less stable transcription when compared to other internal control genes [29]. More recently, the geNorm approach was used to determine the most stably expressed genes in peripheral blood mononuclear cells (PBMCs) of PM and control males [30], to determine the ‘optimal’ method for *FMR1* mRNA normalisation. The *EIF4A2* and *SDHA* mRNA average was found to be the optimal normalisation method. This normalisation method was then applied to PM and control females, where correlations to the phenotype were also investigated [30].

This study expands on the previous findings by examining: (i) how the choice of the normalisation method impacts the strength of the previously published correlation between *FMR1* mRNA level and CGG triplet expansion size in PM females and an age and IQ matched group; (ii) the variability in internal control gene mRNA levels including *EIF4A2*, *SDHA* and *GUS* when compared between PM females and an age and IQ-matched control group; (iii) relationships between CGG triplet expansion size and the mRNA levels of the chosen internal control genes.

## Materials and methods

### Participants

All study participants provided signed informed consent and the study procedures were consistent with the Declaration of Helsinki and approved by the Southern Health Ethics Committee (project 10147B). Participants included 35 PM females and 35 age- and IQ-matched control females recruited as part of previous studies [30]. Groups were matched on height, body mass index (BMI), age and Wechsler Abbreviated Scale of Intelligence (WASI) Full Scale IQ score (see details in [30]). Participants were English speaking with no history of epilepsy or of a serious head injury and had normal (or corrected) vision and hearing, and no sign of colour blindness or intellectual disability (as assessed using the WASI Full Scale IQ score).

### CGG triplet expansion sizing

Four millilitres of venous blood were collected in ethylene diaminetetraacetic acid (EDTA_ tubes (BD, worldwide) from all participants. DNA was extracted using the BIO ROBOT M48 DNA Extractor (Qiagen Inc., Hilden, Germany). The CGG sizing was performed using the Asuragen AmplideX™ *FMR1* Polymerase chain reaction (PCR) Kit, as per manufacturer’s recommendations (Asuragen: Austin, TX, USA) [31].

### RNA extraction and mRNA analysis

One million peripheral blood mononuclear cells (PBMCs) were isolated per participant from venous blood using Ficoll gradient separation as previously described [7], with PBMC pellets frozen at -80°C in RLT buffer for total RNA extraction. Total RNA was purified using the RNeasy extraction kit, as per manufacturer’s instructions (Qiagen Inc., Hilden, Germany). NanoDrop ND-1000 Spectrophotometer was used to determine RNA concentrations in triplicate, with purity assessed using the A260/A280 ratio (expected values between 1.8 and 2). Each RNA sample was then diluted to 5 ng/μl, with 2 μl RNA added for cDNA synthesis performed using the Multiscribe Reverse Transcription System (20 μl total), 50 units/μl of the reverse transcription enzyme (Life Technologies, Global).

Real-time quantitative PCR (RT-PCR) was performed on a ViiA™ 7 System (Life Technologies, Global) to quantify *FMR1*-5′, *FMR1*-3′ and internal control genes (i.e., *GUS*, *EIF4A2* and *SDHA*) using the relative standard curve method, as previously described [30]. *FMR1*-5′, *FMR1*-3′ and *GUS* primers and probes were used at concentrations of 18 μM and 2 μM, respectively, with previously published sequences for RT-PCR primers and probes for: *FMR1*-5′ [10], *FMR1*-3′ [32] and *GUS [10]* assays. Specifically, these sequences included: (i) *FMR1*-5′ forward primer 5′-GCAGAT TCCATTTCATGATGTCA-3′; *FMR1*-5′ reverse primer 5′-ACCACCAACAGCAAGGCT CT-3'; and *FMR1*-5′ probe 5′-(FAM)-TGA TGA AGT TGA GGT GTA TTC CAG AGC AAA TGA-(TAMRA)-3′; (ii) *FMR1*-3′ forward primer 5′-GGAACAAAGGACAGCATCGC-3′; *FMR1*-3′ reverse primer 5′-CTCTCCAAACGCAACTGGTCT-3′; *FMR1*-3’ probe 5′-(FAM)-AATGCCACTGTTCTTTTGGATTATCACCTGAA-(TAMRA)-3′; (iii) *GUS* forward primer 5′-CTCATTTGGAATTTTGCCGAT T-3′; *GUS* reverse primer 5′-CCGAGTGAAGATCCC CTTTTTA-3′; *GUS* probe 5′-(FAM)-TGAACAGTCACCGACGAGAGTGCTGG-(TAMRA)-3′. The *FMR1-5’*and 3’assays target *FMR1* exon 3 /4 and exon 13/14 junctions, respectively. These same assays are mRNA specific (do not amplify DNA), targeting conserved regions of *FMR1* mRNA that are not subject to alternative splicing, as described previously [10, 32, 33].

*EIF4A2* and *SDHA* primer/probe mixes were obtained from PrimerDesign (PerfectProbe ge-PP-12-hu kit) and used at concentration of 2 μM, with sequences not disclosed by the manufacturer. The *FMR1*-5′ and *FMR1*-3′ target gene and the internal control gene dynamic linear range (DLR) common to all the assays was determined to be 1 to 40 ng/μl total RNA input in a 20 μl cDNA reaction. This was determined from a series of doubling dilutions of RNA (160–0.5 ng/ul) of a selected control PBMC sample. All assays showed optimal performance within the DLR, with PCR efficiency ranging between 92 and 94% and coefficient of correlation of greater than 0.98. For all assays in this study, samples were quantified in arbitrary units (au) in relation to the standard curves performed on each plate and had to be within the DLR to be included in further analyses. The mean *FMR1* 5’and 3’ mRNA levels was normalised to: (i) *GUS* alone (*FMR1*/*GUS*); (ii) mean of *EIF4A2* and *SDHA* mRNA levels (*FMR1*/2IC); (iii) mean of *GUS*, *EIF4A2* and *SDHA* mRNA levels (*FMR1*/3IC). *GUS* mRNA levels were normalised to 2IC (*EIF4A2* and *SDHA*); while *EIF4A2* and *SDHA* mRNA was expressed as a ratio (*EIF4A2/SDHA)*.

Two separate cDNA reactions were performed for each RNA sample, with each cDNA analysed in two separate RT-PCR reactions. The summary measure for mRNA level for each participant was represented by the mean of the four outputs. *FMR1* mRNA results were not obtained for two PM females and two control females from the 70 participants because there was either insufficient RNA extracted or because the results failed the 5′ and 3′ *FMR1* mRNA quality control step [33].

### Statistical analyses

The Shapiro-Wilk normality test was used to check normal distribution for each of the *FMR1* mRNA datasets, separately for each group. The data was then transformed if normality was not achieved. *FMR1*/*GUS* data was transformed using a natural logarithm function while for all other data, reciprocal function was used The Generalised estimating (GEE) method was then used for the inter-group comparison, taking into account correlation within family in the PM cohort. For the relationship between each mRNA level and CGG size, piecewise linear regression was used to find a threshold, resulting in two different slopes, above and below the threshold. Analyses were carried out using STATA software. See S1 Table for raw data.

## Results

### Intergroup comparisons of *FMR1* mRNA levels between PM and control groups

**Fig 1:**
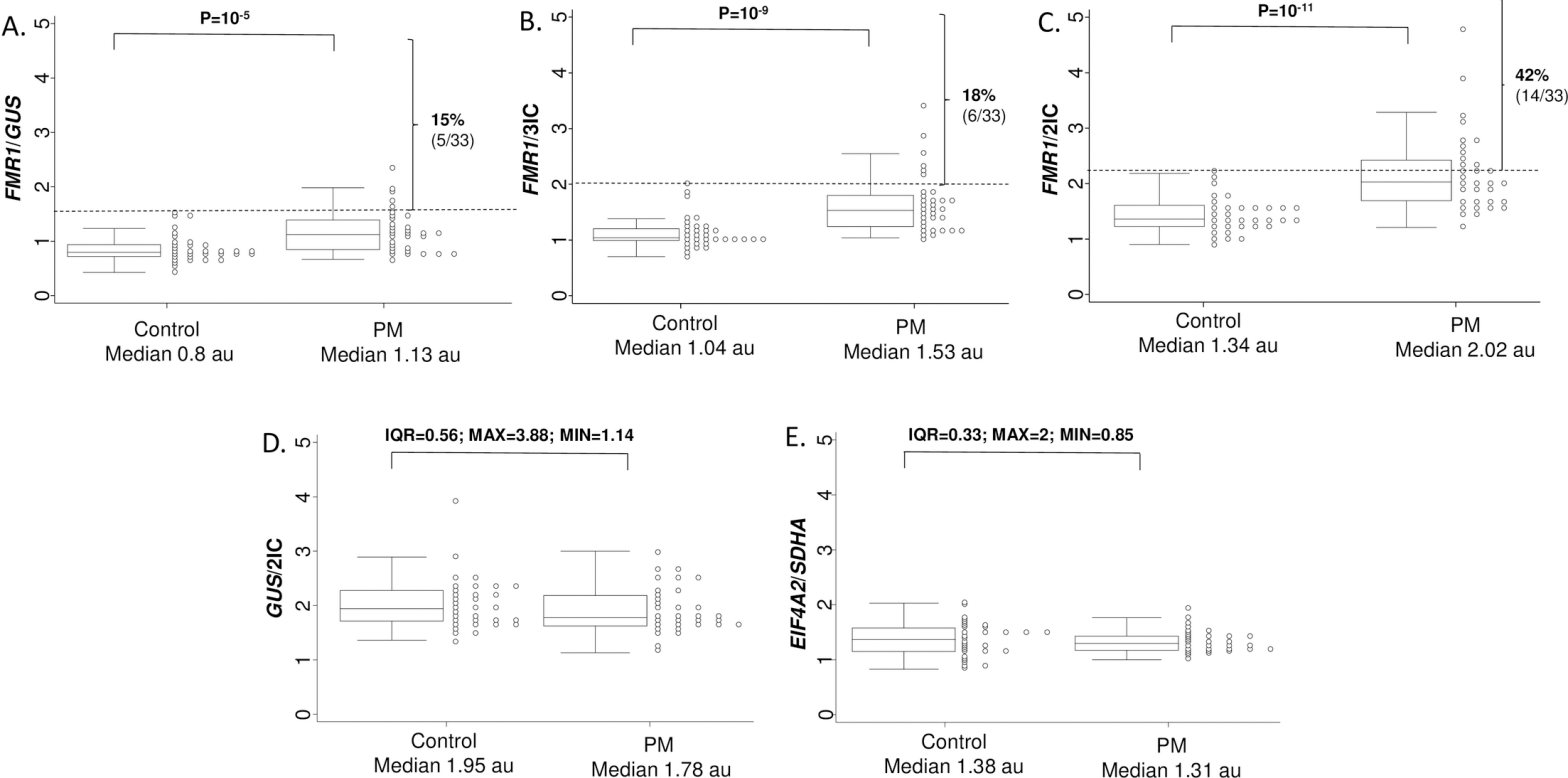
Inter-group mRNA level comparison plots between 33 PM and 33 control females. *FMR1* mRNA was normalised to either **(A)** *GUS* alone; **(B)** 3IC (*GUS*, *EIF4A2* and *SDHA*); or **(C)** 2IC (*EIF4A2* and *SDHA*, without *GUS*). **(D)** *GUS* mRNA levels were normalised to 2IC (*EIF4A2* and *SDHA*). **(E)** Variability in *EIF4A2* to *SDHA* mRNA ratio (*EIF4A2* and *SDHA)*, between groups is also presented. **Note:** Broken horizontal lines indicate the maximum control value for each plot with percentages above this line indicating the proportion of PM females with abnormally increased *FMR1* mRNA levels. Control and PM CGG groups reflect range in P values correspond to Table 1 (30). Interquartile range (IQR); maximum value (MAX); minimum value (MIN).

Three different methodologies for normalisation of *FMR1* mRNA levels in blood were compared between PM and control groups: *FMR1*/*GUS*, *FMR1*/3IC and *FMR1*/2IC. *FMR1* mRNA levels were significantly elevated in the PM group compared to the control group for all three normalisation approaches (*FMR1*/*GUS*: *p* = 2.7×10^−5^; *FMR1*/3IC: *p* = 1.2×10^−9^; *FMR1*/2IC: *p* = 3.4×10^−11^). However, the most significant difference in mean values between the two groups was observed using the *FMR1*/2IC approach (that does not use *GUS* mRNA for normalisation) (Fig 1). The choice of normalisation strategy also influenced the proportion of PM females that exceeded the maximum control value for each plot (Fig 1: broken horizontal lines), with only 5 exceeding this value for *FMR1*/*GUS* (15%) and 6 for *FMR1*/3IC (18%) compared to 14 females for *FMR1*/2IC (42%) where *GUS* had been omitted from the normalisation equation. *GUS/*2IC and *EIF4A2/SDHA* values were not significantly elevated in the PM group compared to controls (Fig 1D and 1E; Table 1). However, the *GUS/*2IC value showed much greater variability between individuals with interquartile range twice as large in both control and PM groups as compared to the *EIF4A2/SDHA* value.

**Table 1:** Relationships between *FMR*1 mRNA normalised using three different methods and CGG repeat size in PM females.

Outcome	Predictor	Coef	s.e	*p*-value
***FMR1*/*GUS***	CGG: Threshold = 79; 95% CI = (64, 93)			
	CGG ≤ 79	0.20	0.19	0.287
** **	CGG > 79	2.05	0.59	0.001
***FMR1*/3IC**	CGG: Threshold = 83; 95% CI = (73, 93)			
	CGG ≤ 83	0.49	0.20	0.017
** **	CGG > 83	3.25	0.723	3.0 x 10^−5^
***FMR1*/2IC**	CGG: Threshold = 86; 95% CI = (78, 94)			
	CGG ≤ 86	0.82	0.23	0.001
	CGG > 86	5.07	0.95	1.3 x 10^−6^
***GUS*/2IC**		0.09	0.17	0.617
***EIF4A2*/*SDHA***		0.04	0.12	0.747

**Note:** Piecewise linear regression was used to find a threshold, resulting in two difference slopes above and below the threshold.

Estimated regression coefficient (Coef) and standard error (s.e) were multiplied by 100. 3IC = normalisation by *GUS*, *EIF4A2* and *SDHA*; 2IC = normalisation by *EIF4A2* and *SDHA*, without *GUS*.

### Relationships between mRNA levels and CGG triplet repeat size in PM and control female samples

**Fig 2:**
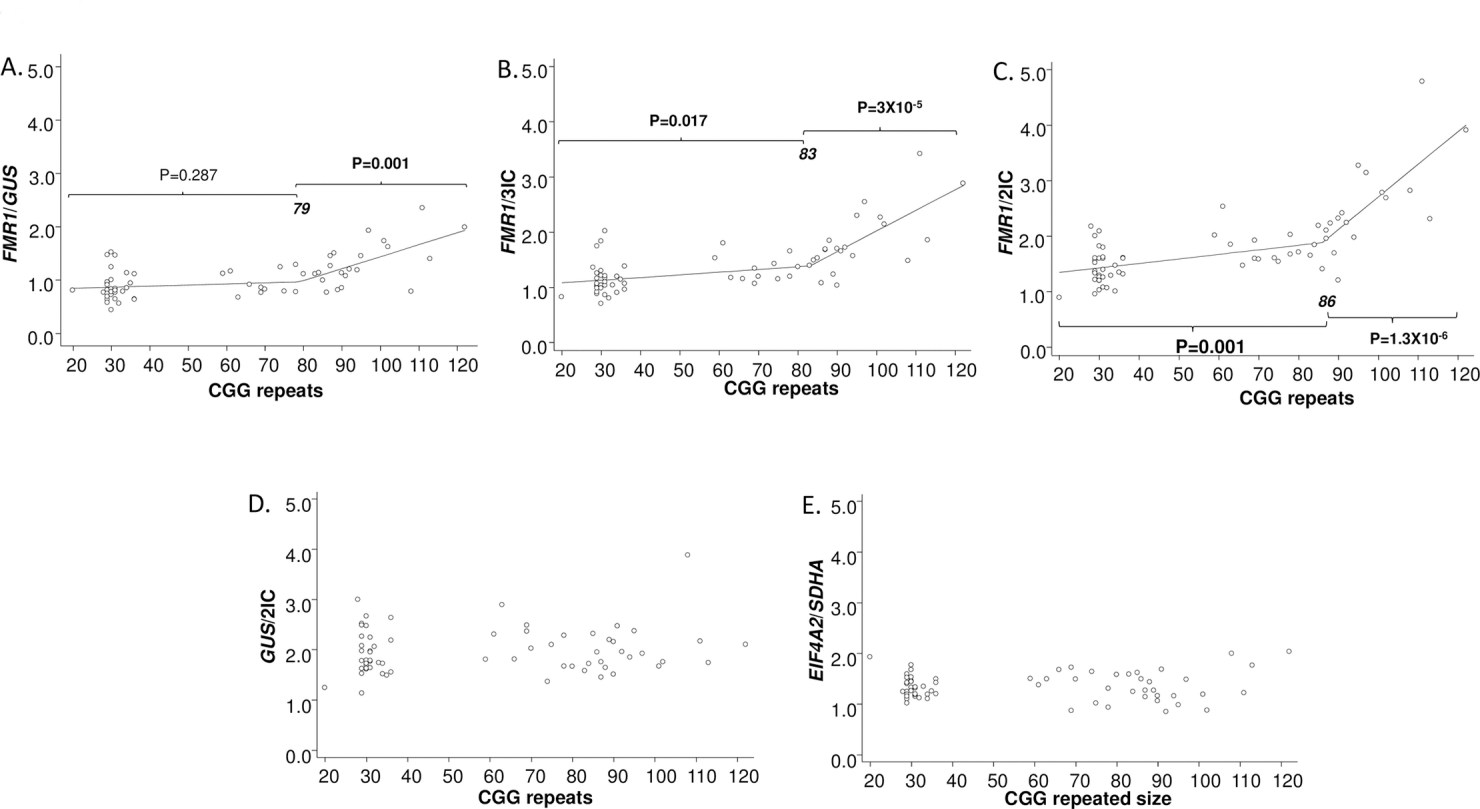
Relationship between CGG triplet repeat size and mRNA levels in the combined cohort of 33 PM and 33 control females. CGG size versus *FMR1* mRNA normalised to either **(A)** *GUS* alone; **(B)** 3IC (*GUS*, *EIF4A2* and *SDHA*); **(C)** 2IC (*EIF4A2* and *SDHA*, without *GUS*). CGG size versus **(D)** *GUS* mRNA levels were normalised to 2IC (*EIF4A2* and *SDHA*); **(E)** *SDHA*/*EIF4A2* mRNA ratio variability between groups is also presented. **Note:** Piecewise linear regression was used to find a threshold in A, B and C, resulting in two difference slopes above and below the threshold (the CGG repeat threshold is presented in bold italics). The regression coefficients and standard errors for these relationships are described in Table 1.

The influence of *FMR1* normalisation approach on *FMR1* mRNA-CGG relationships in PM and control groups was assessed. In the combined cohort of PM and control females, piecewise linear regression was used to find a threshold CGG repeat size where two different slopes could be differentiated. Below this threshold, CGG repeat size was not significantly correlated with *FMR1*/*GUS* data (*p* = 0.287), but was significantly correlated with data for *FMR1*/3IC (*p* = 0.017) and *FMR1*/2IC (*p* = 0.001). The strength of the relationship also varied above the threshold depending on the *FMR1* normalisation approach that had been used. In particular, effect size of the *FMR1* mRNA-CGG relationship was higher with a smaller *p*-value for *FMR1*/2IC (regression coefficient (*β*) = 5.07, *p* = 1.3 × 10^−6^) and *FMR1*/3IC (*β = 3*.*25*, *p* = 3 × 10^−5^) than it was for *FMR1*/*GUS* (*β = 2*.*05*, *p* = 0.001). These analyses demonstrate that removal of *GUS* from *FMR1* mRNA normalisation and/or dilution of the *GUS* contribution by addition of the two other internal control genes improves the *FMR1* mRNA-CGG size relationship and also increases the slope for this relationship. In contrast, *GUS/*2IC and *EIF4A2/SDHA* values were not significantly correlated with CGG size (Fig 2D and 2E; Table 1). *GUS/*2IC value also showed greater variability between individuals across the full range of CGG repeat size (from control to PM) as compared to the *EIF4A2/SDHA* value.

## Discussion

This study demonstrates that correlation between *FMR1* mRNA levels and CGG triplet repeat size is weakened when *GUS* is used as an internal control gene for mRNA analysis studies in PBMCs of PM females. Furthermore, using *GUS* mRNA as a sole control in the denominator led to an underestimation of *FMR1* mRNA levels for PM females when compared to age-matched controls. Stronger correlations were observed between *FMR1* mRNA and CGG triplet repeat size with the slope of the relationship increased when *FMR1* mRNA levels were normalised without *GUS* or when contributions from *GUS* were minimised by inclusion of the two internal control genes *EIFA2* and *SDHA*.

PM *GUS* mRNA levels (*GUS*/2IC) were significantly correlated with gait and verbal intelligence scores in PM but not control females [30]. However, in this study the *GUS*/2IC values: (i) did not significantly correlate with increased CGG triplet repeat size, and (ii) did not significantly differ between control and PM groups. *GUS*/2IC values were found to be more variable than the *EIF4A2/SDHA* ratio values in both PM and control groups between individuals. This suggested that *GUS* mRNA could be confounding if used to normalise *FMR1* mRNA levels in RT-PCR experiments (Figs 1 and 2). This is consistent with the earlier stability study of mRNA levels for a panel of genes using the geNorm approach in another cohort [30]. This study showed that *EIF4A2* and *SDHA* mRNA levels were more stable than that of *GUS* in control and PM males (Supplementary Figure 1 in [30]).

In this study, *FMR1* mRNA level normalised to *GUS* alone resulted in an underestimation of *FMR1* mRNA in samples that had high levels of *GUS*/2IC mRNA. On the other hand, for samples with very low levels of internal control gene mRNA, *FMR1* levels were over-estimated. The inter-individual variability in *GUS* mRNA level detected in blood in both the control and PM groups could be due to biological or environmental factors that directly or indirectly influence the activity of the *β*-glucuronidase enzyme [34, 35]. Furthermore, *GUS* mRNA stability level has been reported to be influenced by gender, age and cell type [36–38]. This limitation has been partly addressed here through the use of different internal control genes (i.e., *EIF4A2* and *SDHA*)

Normalisation of *FMR1* mRNA levels in PM and control PBMCs with *EIFA2* and *SDHA* (*FMR1*/2IC) has been demonstrated in previous studies examining relationships between *FMR1* mRNA level and phenotype [39–41]. Specifically, in twenty PM females without FXTAS, higher *FMR1*/2IC values correlated with mean diffusivity within the middle cerebellar peduncle determined by diffusion-weighted imaging. *FMR1*/2IC value was also significantly correlated with poor performance on the Paced Auditory Serial Addition Test scores indicating executive dysfunction and/or slow processing speed [39].

In summary, these findings demonstrate that *GUS* normalisation in PBMC studies masks the relationship between *FMR1* mRNA level and CGG triplet repeat size. It also artificially decreases values for *FMR1* mRNA level in PBMC PM data, as compared to values normalised with *EIF4A2* and *SDHA* levels. These findings may not apply to other tissue types, *FMR1* alleles other than PM or other age groups. Instead, the most stable combinations of optimal internal control genes should be determined for each setting separately, using validated approaches such as geNorm [42]. This is consistent with the fact that there are no ideal internal control genes across all settings. This is now accepted widely in other fields of genetics [29, 43] and should be implemented for investigations of Fragile X-associated disorders. Utility of absolute quantification of mRNA determined through methods that do not rely on internal control normalisation, including competitive PCR [44] and Droplet Digital PCR [45], may circumvent this problem in future studies.

## Supporting information

S1 Table *FMR1*, *GUS*, *SDHA* and *EIF4A2* mRNA data obtained using relative standard curve real-time PCR method from peripheral blood mononuclear cell RNA of 33 PM and 33 control females. (XLSX)
